# Structures of an Apo and a Binary Complex of an Evolved Archeal B Family DNA Polymerase Capable of Synthesising Highly Cy-Dye Labelled DNA

**DOI:** 10.1371/journal.pone.0070892

**Published:** 2013-08-05

**Authors:** Samantha A. Wynne, Vitor B. Pinheiro, Philipp Holliger, Andrew G. W. Leslie

**Affiliations:** Medical Research Council Laboratory of Molecular Biology, Cambridge, United Kingdom; Institute of Molecular Genetics IMG-CNR, Italy

## Abstract

Thermophilic DNA polymerases of the polB family are of great importance in biotechnological applications including high-fidelity PCR. Of particular interest is the relative promiscuity of engineered versions of the exo- form of polymerases from the *Thermo-* and *Pyrococcales* families towards non-canonical substrates, which enables key advances in Next-generation sequencing. Despite this there is a paucity of structural information to guide further engineering of this group of polymerases. Here we report two structures, of the apo form and of a binary complex of a previously described variant (E10) of *Pyrococcus furiosus* (Pfu) polymerase with an ability to fully replace dCTP with Cyanine dye-labeled dCTP (Cy3-dCTP or Cy5-dCTP) in PCR and synthesise highly fluorescent “CyDNA” densely decorated with cyanine dye heterocycles. The apo form of Pfu-E10 closely matches reported apo form structures of wild-type Pfu. In contrast, the binary complex (in the replicative state with a duplex DNA oligonucleotide) reveals a closing movement of the thumb domain, increasing the contact surface with the nascent DNA duplex strand. Modelling based on the binary complex suggests how bulky fluorophores may be accommodated during processive synthesis and has aided the identification of residues important for the synthesis of unnatural nucleic acid polymers.

## Introduction

Sequence analysis has identified six different families of DNA-dependent DNA polymerases (A, B, C, D, X and Y) [1], [2]. Family A includes DNA polymerases from *Thermus aquaticus*, *Bacillus stearothermophilus*, *Escherichia coli* (DNA pol I), and T7 bacteriophage, whilst the replicative DNA polymerases from eukaryotes and archaea, and bacteriophages RB69 and T4, belong to the B family. Family C polymerases include the main eubacterial replicating polymerase, PolIIIα, Family D includes euryarchaeal hetero-dimeric DNA polymerases [3], and X and Y family polymerases are involved in DNA repair and lesion bypass [1], [2]. High resolution structures of apo, binary and ternary complexes have been obtained for KlenTaq (polA) [4], [5], RB69 polymerase (polB) [6], [7], *E. coli* polIII (polC) [8], [9], Dpo4 (polY) [10], and polbeta (polX) [11]. However, while there are several high-resolution structures of the apo-forms of biotechnologically important polB family polymerases from hyperthermophilic archea (*Thermococcus gorgonarius* (Tgo) [12], *Thermococcus sp. 9_N-7* (9°N) [13], *Pyrococcus kodakaraensis* KOD1 [14], *Pyrococcus furiosus* Pfu [15], and editing complexes of Tgo [16] and *Pyrococcus. abyssi* [17]) there are no reported structures of binary or tertiary complexes of the replicative state.

The replicative DNA polymerase from the hyperthermophilic archaeon *Pyrococcus furiosus* (Pfu) is a member of that polB family and is used extensively in biotechnology applications including high-fidelity PCR. Many other applications rely on the incorporation of fluorescent dye labelled nucleotides. However, although readily incorporated at low levels (e.g. 10%) of substitution, dye labelled nucleotides are poor polymerase substrates at high or full substitution due to the large size of the fluorophore substituent and cause stalling of the wild type polymerase after just a few incorporation steps. We had previously used polymerase evolution by short patch compartmentalised self-replication (spCSR) [18] to discover variants of Pfu with a dramatically enhanced ability to incorporate Cy3- and Cy5-labelled dCTP and replicate Cy-dye labelled DNA. The resulting variant E10, derived from an exonuclease-deficient version of Pfu polymerase (with mutations V93Q, D141A and E143A) and with additional mutations in the conserved A- (V337I, E399D, N400D, R407I) and C- active site sequence motifs (Y546H) was able to efficiently PCR DNA while completely replacing all dCTP on both strands with cyanine labelled dCTP (Cy3-dCTP or Cy5-dCTP) giving rise to highly-fluorescent products, termed Cy-DNA [19] with applications in advanced microscopy applications [20].

The creation of other variants of the polB family of DNA polymerases have enabled key advances due to an expanded substrate spectrum. These include “Therminator™” and 9°N DNA polymerase (A485L) [21] as well as other 9°N DNA polymerase variants with mutations in the vicinity of residue 485, which have important applications in Next-generation sequencing technologies (Illumina patent. [22]). Other mutations in the thumb domain of the closely related *T. gorgonarius* (Tgo) DNA polymerase enable processive synthesis of artificial genetic polymers (XNAs) [23] and RNA [24]. However, progress in the further design, engineering and evolution of polymerases for these and other applications is hindered by the absence of high quality structural data on thermophilic polB polymerases. In an attempt to better understand the molecular basis for the altered substrate specificity of Pfu E10 polymerase and generally gain a better structural understanding of the primer-template duplex recognition in this class of DNA polymerases, we have solved the crystal structures of the apo Pfu-E10 and a Pfu-E10:DNA binary complex at 2.4 Å and 2.9 Å resolution respectively and compare them to previously obtained structures.

## Results and Discussion

### CyDNA Synthesis by the E10 Polymerase

**Figure 1:**
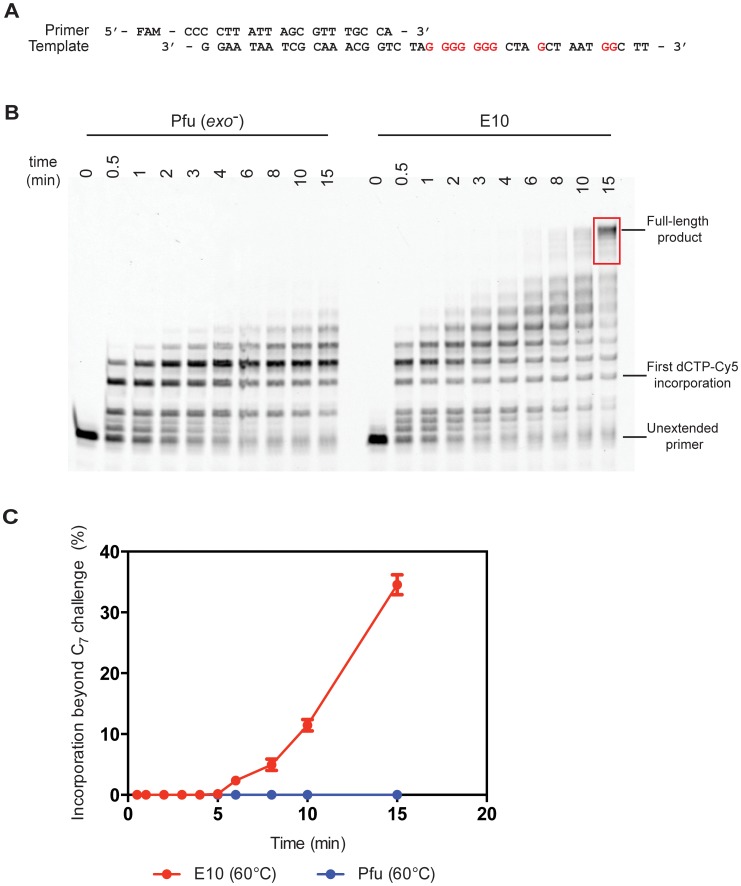
Polymerase activity at 60°C. A) The primer extension assays used a primer labelled with FAM (fluorescein) and unlabelled template. Sites of Cy5-dCTP incorporation (Gs in the template) are shown in red. B) Primer extension time course comparing wild-type Pfu(exo-) and engineered Pfu-E10 polymerases at 60°C. Extension times are shown in minutes. Extension products used to quantify extension beyond the seven consecutive Cy5-dCTP incorporations (C_7_ challenge) are highlighted in red – see Materials and Methods for details. C) Fraction of the primers extended beyond the C_7_ challenge for both tested polymerases – results are shown for two independent experiments.

Although many natural polymerases are capable of efficient incorporation of and extension from a single Cy3 or Cy5-modified nucleotide, the synthetic challenge rapidly escalates at higher incorporation densities due to the steric bulk (and potentially other effects) of the large, hydrophobic cyanine dye heterocycles clustering in the major groove. This has hindered synthesis of DNA with high-density arrays of Cy-dyes and the applications this might enable and has spurred the development of a dedicated polymerase Pfu-E10 [19], [20]. Indeed, primer extension assays from challenging templates (that require multiple consecutive incorporations of the modified nucleotide) demonstrate the extent to which the evolved polymerase (Pfu-E10) outperforms the wild-type (Pfu(exo-)) polymerase (Figure 1, Figure S1). Here the primer is labelled with fluorescein amidite (FAM) so that it can be visualised regardless of cyanine dye incorporation. In both conditions, the engineered polymerase Pfu-E10 readily synthesises full-length product traversing a dG_7_ template stretch requiring the consecutive incorporation of seven Cy5-dCTPs within the experimental time frame. In contrast, the wild-type Pfu exo- polymerase cannot traverse that stretch and stalls after incorporation of maximally five consecutive Cy5-dCTPs (with a major pause at n+3).

**Figure 2:**
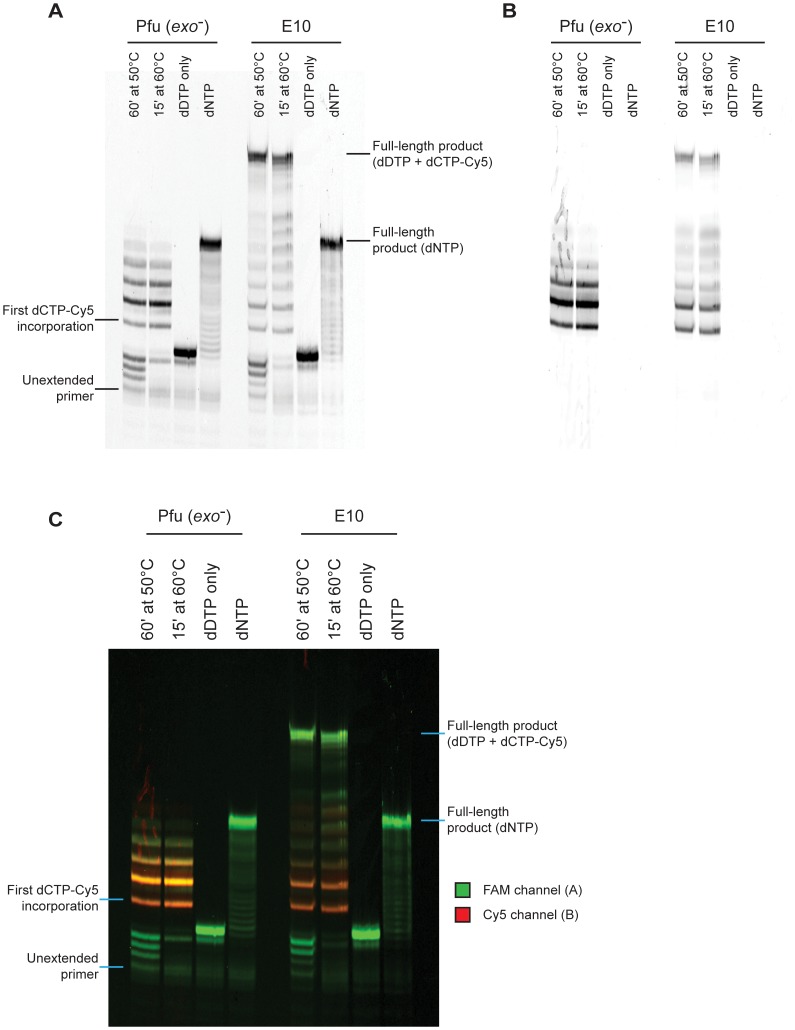
Summary of primer extension assays. A) Key primer extension reactions visualised via the FAM channel. Only engineered E10 polymerase is capable of extending the FAM-labelled primer to full-length when incorporating Cy5-dCTP. No template-independent extension is observed in the absence of dCTP (dDTP only ie dGTP, dTTP and dATP) and both enzymes can synthesise the template with the natural dCTP substrate (dNTP). The discrepancy in migration between primers extended with natural triphosphates and with Cy5-dCTP, is due to the significant increase in monomer mass introduced by the Cy5 moiety. B) The same reactions visualised by the Cy5 channel to see only Cy5-labelled primer. It is clear that Cy5 signals are only observed after the incorporation of a Cy5-dCTP and co-localise to the signals observed in the FAM channel. C) Overlay of A and B in colour.

Primer extension was strictly dependent on the presence of a full contingency of nucleotides: no extension was observed (for either E10 or Pfu exo-) when either dCTP or Cy5-dCTP were omitted from the reaction mix (Figure 2A). Incorporation of Cy5-dCTP could be demonstrated both by the changes in PAGE electrophoretic mobility (with Cy5-dCTP migrating slower than its natural counterpart due to the large mass of the hydrophobic cyanine dye heterocycle) and by monitoring Cy5 fluorescence (Figure 2B,C).

### Structure of Apo Pfu-E10 Polymerase

**Figure 3:**
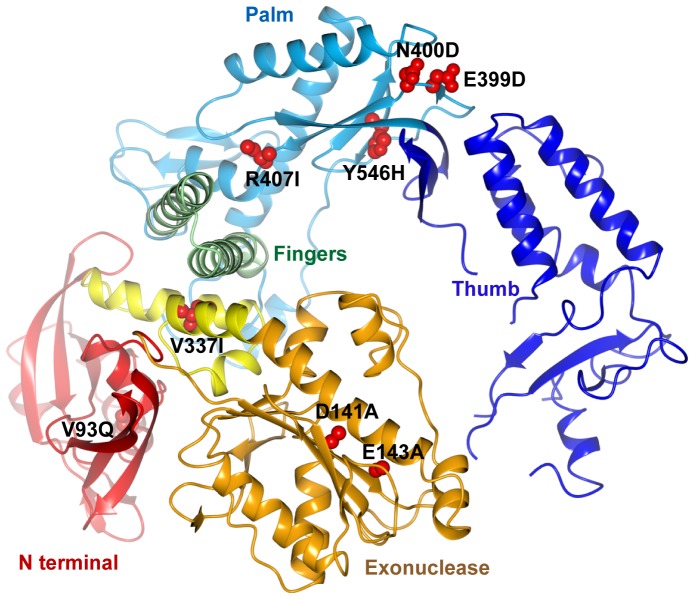
Structure of the Pfu-E10 polymerase. Cartoon representation of the apo polymerase structure with domains coloured as follows: N-terminal domain: red, exonuclease domain: gold, linker region: yellow, palm domain: pale blue, fingers domain: green and thumb domain: dark blue. The side chains of the mutated residues (V93Q, D141A, E143A, V337I, E399D, N400D, R407I and Y546H) are shown with atoms represented as red spheres.

The 2.4 Å resolution structure of apo Pfu-E10 was solved by molecular replacement using the structure of Tgo DNA polymerase (PDB code 1TGO) [12] as the search model. Data processing and refinement statistics are shown in Table 1. Pfu-E10 displays the domain structure characteristic of DNA polymerases (Figure 3), consisting of an N-terminal domain (residues 1–130), an exonuclease domain (131–326), a linker region (327–369), a palm domain (370–450 and 501–586), a fingers domain (451–500) and a thumb domain (587–775). The electron density is well defined for the N-terminal, exonuclease, linker, palm and fingers domains of both molecules in the crystallographic asymmetric unit, but some parts of the thumb domains are disordered. The final model for chain B is the most complete, but is missing amino acids 612–614, 669–675, 693–695 and 757–775. The two molecules (chains A and B) are very similar (rmsd of 0.8 Å for 690 alpha carbon atoms). There is a slight difference in the orientation of the thumb domain, which has a slightly more open conformation in chain B than chain A. This is likely to be due to the different crystal contacts in this region.

**Table 1:** Data collection and refinement statistics.

Polymerase	Apo Pfu-E10	Pfu-E10:DNA
PDB identifier	4AHC	4AIL
**Cell Parameters**		
Space group	P2_1_	C222_1_
*a, b, c* (Å)	78.4 197.9 78.4	92.9 126.8 205.6
a, b, g (°)	90.0 108.4 90.0	90.0 90.0 90.0
Twin law	*l –k h*	n/a
Twinning fraction	0.46	n/a
**Data Collection**		
Resolution range (Å)	41.20–2.4	60.52–2.9
No of unique reflections^1^	78466 (10690)	26919 (3865)
Completeness (%)^1^	89.1 (83.0)	98.8 (98.9)
Multiplicity^1^	2.7 (2.3)	3.6 (3.7)
Rmerge^1^	0.075 (0.471)	0.084 (0.727)
Mean I/s(I) 1	9.6 (2.1)	10.6 (2.0)
**Refinement Statistics**		
Resolution range (Å)	98.5–2.4	102.8–2.9
R work^1^	0.207 (0.287)	0.227 (0.364)
R free^1^	0.231 (0.345)	0.259 (0.388)
Ramachandran outliers	1	0
rmsd bond lengths (Å)	0.004	0.013
rmsd bond angles (°)	0.76	0.83
**Number of Atoms/Residues**		
Protein	11863	6145
Nucleic acid	0	16 bases (329 atoms)
Glycerol molecules	4	0
Waters	294	79

1 Values in parentheses are for the highest resolution bin (2.53–2.40 Å and 2.46–2.40 Å for the apo Pfu-E10 for scaling and refinement respectively, with 2.98–2.9 Å and 3.06–2.90 Å for the Pfu-E10:DNA complex).

During this work the crystal structures of native Pfu polymerase (Pfu-pol, PDB ID 2JGU) [15] and of the complex with proliferating cell nuclear antigen (Pfu-PCNA, PDB ID 3A2F) [25] were determined at resolutions of 2.6 Å and 2.7 Å respectively. The structure of apo Pfu-E10 is very similar to both these structures, with a superposition rmsd of 1.0 Å and 0.9 Å between Pfu-pol and Pfu-E10 (molecule A or B respectively, 685 residues aligned), and 1.0 Å rmsd between the Pfu-PCNA and Pfu:E10 Chain A (694 residues aligned) and 1.5 Å for Chain B (715 residues aligned). The greatest difference between the three models is in the thumb domain, especially in the region of residues 679–730. The electron density for this region is poorly defined for Pfu-E10, there are missing residues in all three models, and the refined temperature factors are very high (typically >90 Å^2^), suggesting a significant level of structural flexibility in the absence of bound DNA. There is no evidence of any conformational changes resulting from the mutations in Pfu-E10.

Overall, the structure of the Pfu-E10 polymerase is very similar to other archaeal polymerase structures, (rmsd values of 1.7–2.7 Å, Table 2). This is unsurprising given the high sequence identity (81%) between Pfu-E10 and Tgo [12] or 9°N polymerases [13]. Non-archaeal B family DNA polymerases, such as RB69 DNA polymerase [6], are less similar with an rmsd of 3.3 Å between Pfu:E10 and apo RB69 polymerase, although the rmsd values are significantly smaller when individual domains are superimposed.

**Table 2:** Structural similarity of Apo Pfu-E10 with other polymerases.

Comparison with Pfu-E10 Molecule B	Residues aligned	RMSd^1^	% identity^2^
Apo A	690	0.8	N/A
Wild-type Pfu	685	0.9	N/A
Pfu-E10:DNA complex	654	1.1	N/A
Wild-type Pfu with PCNA	715	1.5	N/A
Tgo	633	1.7	81
KOD1	671	1.7	80
9Deg North	586	2.7	81
RB69 (Apo)	608	3.3	12
RB69+DNA	560	3.3	12

1 The RMSd was calculated using Superpose from the entire length of the Pfu-E10 except where stated.

2 ClustalW2 was used for obtaining % identity score.

### Structure of Pfu-E10 : DNA Binary Complex

**Figure 4:**
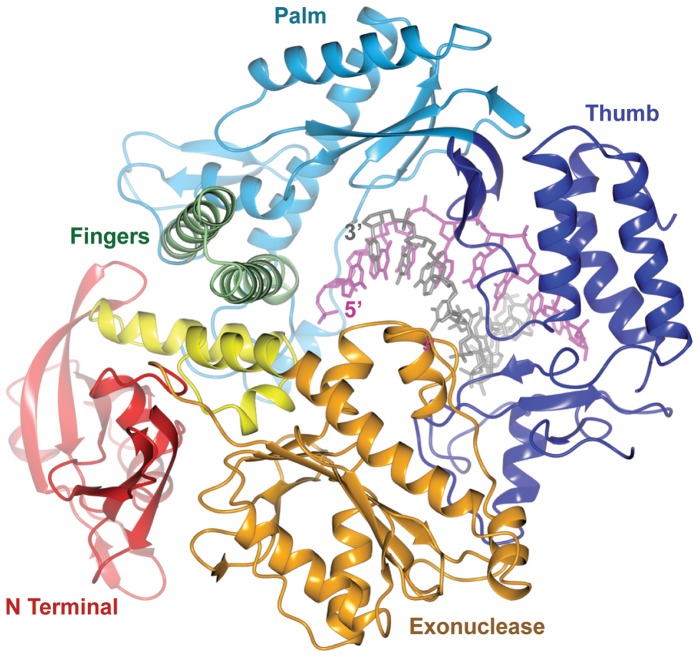
Structure of the Pfu-E10:DNA binary complex. Cartoon representation of the binary complex**.** Polymerase domains are coloured as in Figure 3. The DNA primer strand is shown in grey, the template strand in magenta.

**Figure 5:**
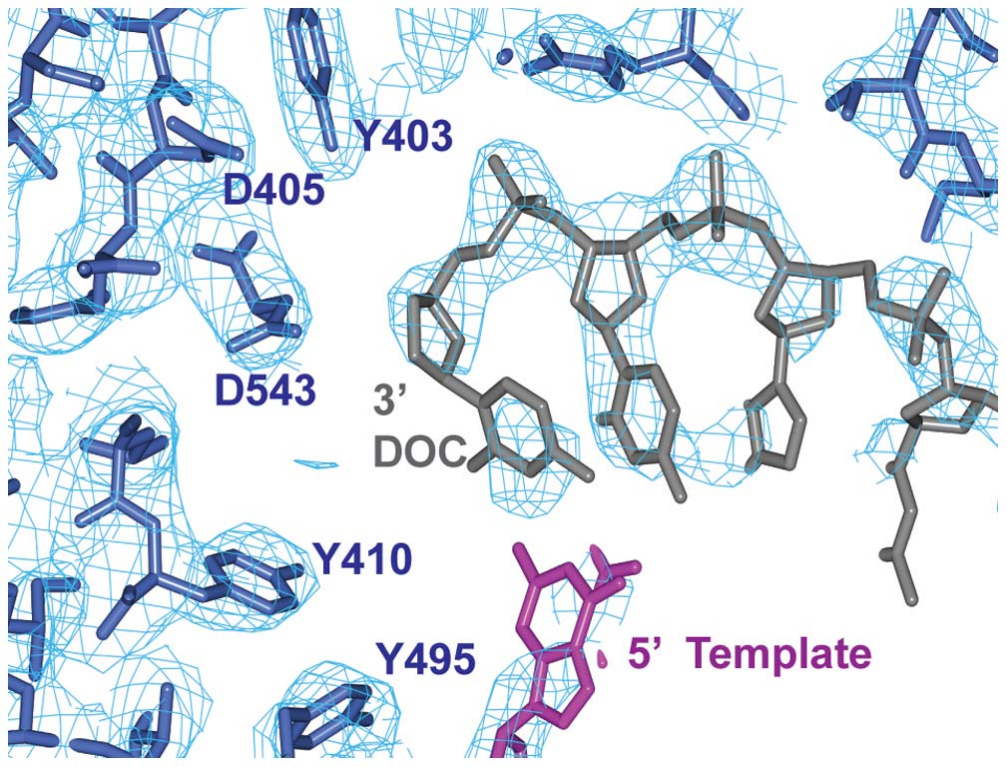
Density for the DNA and active site in the Pfu-E10:DNA binary complex. An omit map was calculated omitting the co-ordinates of the DNA. This map, contoured at 1.25 sigma is shown in pale blue, with the DNA primer and terminal dideoxy cytidine (DOC) in grey, the DNA template in magenta, and Pfu:E10 residues in dark blue.

Pfu-E10 polymerase was crystallised with a primer template duplex of DNA with a 2′–3′ dideoxy terminal cytosine at the 3′ end of the primer to stall the polymerase. The structure of the binary complex (Figure 4) was solved at 2.9 Å resolution using the apo Pfu-E10 structure as the molecular replacement model (Table 1). The presence of the DNA stabilises some parts of the thumb domain and density was visible for three regions that were missing in the model for the apo form (residues 612–614, 669–675 and 693–695). Many of these newly visualised residues interact with the primer strand of the DNA. The density for the double stranded region of the DNA is very clear (template T4-T11 and primer P1-P8, Figure 5), however there is no visible density for the template base (T3) that would bind that incoming nucleotide triphosphate or for template bases T1 and T2.

There is extra density visible at the 5′ end of the primer (remote from the active site) at a lattice contact. It is possible that this could be due to a single molecule of dCTP, stacking against the ends of the DNA, but the density is ambiguous and has not been modelled. However, there is no evidence for a bound nucleotide triphosphate at the active site, in spite of the presence of 10 mM dCTP in the crystallisation medium.

### DNA-protein Interactions

**Figure 6:**
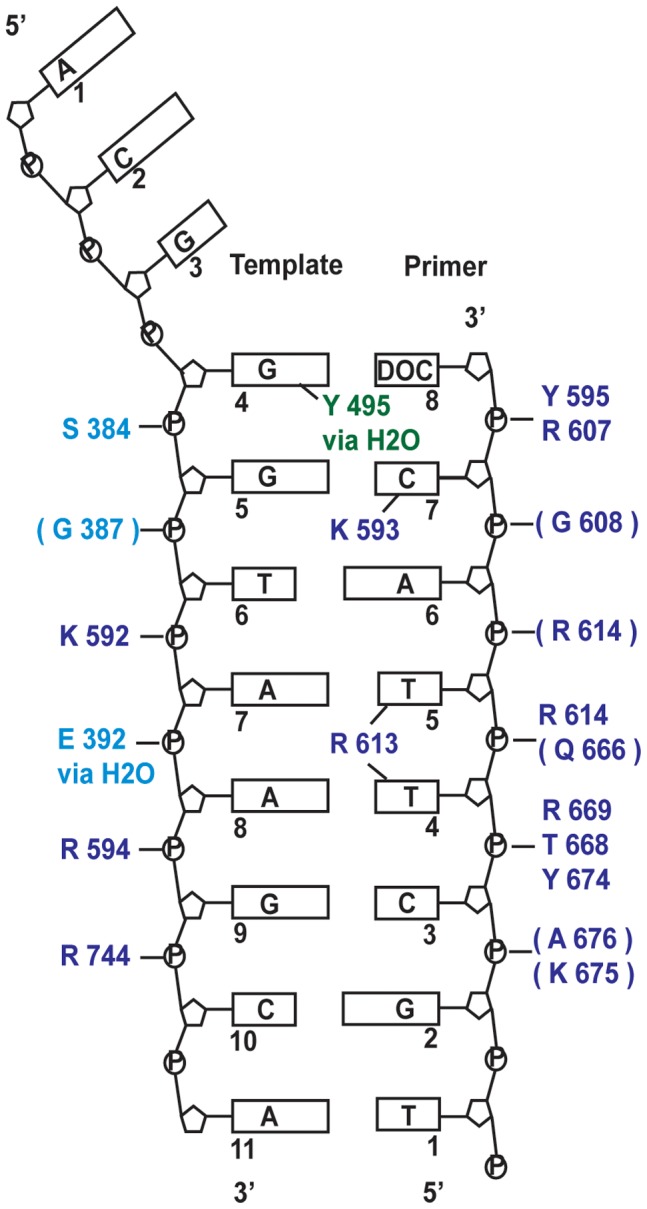
Protein-DNA interactions in the Pfu-E10 binary complex. Schematic representation of the protein DNA-contacts. Brackets indicate that the interaction is via the main chain peptide rather than the side chain, with residues coloured by domain with palm domain: pale blue, fingers domain: green and thumb domain: dark blue. Most interactions are with the sugar-phosphate backbone, however residues in the centre of the figure indicate a minor groove interaction. Full details are given in the text. The three residues at the 5′ end of the template strand could not be modelled.

The primer:template DNA interacts with residues from the palm, fingers and thumb domains of the polymerase (Figure 6). Most interactions are with the sugar-phosphate backbone, either directly, or via bridging water molecules. Not all of the residues are conserved throughout the B family polymerase sequences, but an overall channel of basic residues is maintained that stabilise the negatively charged phosphate groups.

Many DNA polymerases appear to use interactions with the minor groove of newly formed DNA to check for mis-incorporated nucleotides [26]. In correct Watson-Crick geometry, the two hydrogen bond acceptors (N3 of purines and O2 of pyrimidines) in the minor groove would be in approximately the same position regardless of the DNA sequence. The Pfu-E10:DNA binary complex has three residues making minor-groove interactions: Tyr 495, Arg 613 and Lys 593.

Tyr 495 in the fingers domain makes a minor groove interaction via a water molecule with the template base N3 acceptor of the final base pair of the duplex. This tyrosine is totally conserved in B family polymerases. In the event of a base mismatch, Watson-Crick geometry would be absent resulting in the loss of this stabilising interaction and promoting repositioning of the DNA to the editing site of the polymerase. The RB69 residue at this position (Tyr 567) makes a water-mediated H bond to the N3 acceptor of the template guanine of the final primer-template base pair in the ternary structure [7].

The side chain of Arg 613 extends into the minor groove between primer P4 and P5 and could form hydrogen bonds with the O2 acceptors in both primer P4 and P5 thymine bases. A salt bridge between Arg 613 and Asp 615 ensures the correct orientation of the guanidinium group. In Phi29 polymerase [27], the equivalent Lys 555 is too far away from the minor groove to contact it in a binary complex, but does appear to interact via a water molecule in the ternary complex. The RB69 polymerase equivalent, Lys 734, interacts with N3 of template T8 and O2 of the paired primer base via a water molecule in the ternary complex.

A third minor groove interaction involves the side chain of Lys 593 that extends into the minor groove between primer P7 and template T5, forming a hydrogen bond with the O2 acceptor of the penultimate primer P7 cytosine. The lysine side chain is oriented by a salt bridge interaction with Asp541. Equivalent lysine residues in RB69 (Lys 706) and Phi29 (Lys 498) polymerase complex structures interact with the minor groove in the same way. This is the only minor groove contact for Phi29 polymerase.

Lys 593 is within a highly conserved KKRY sequence motif (Pfu-E10 592–595, RB69 705–708) in the thumb domain which is only found in the B family polymerases [1]. It is thought that this KKRY motif stabilises the B form of DNA in RB69 by drawing the template and primer strands closer together [7]. In Pfu-E10 these residues contact both primer and template phosphates. Lys 592 interacts with the phosphate of template T7, Arg 594 makes charge-charge interactions with the phosphate of template T9 and Tyr 595 binds to the final phosphate of the primer (P8).

There are no Pfu-E10 protein-DNA interactions corresponding to those involving Lys 800 and Ala 394 in the RB69 ternary complex. Pfu-E10 Lys 675 (equivalent to RB69 Lys 800) is too distant from the DNA to form an interaction, and there is no evidence at Pfu-E10 Gly 388 for the water molecule that mediates a minor grove contact with the equivalent RB69 Ala 394.

The DNA conformation in the Pfu-E10:DNA binary complex appears to be B form, but due to the high B factors in this region the sugars do not all conform to C3 *exo*, C2 *endo* conformation. The minor groove width near the active site (measured between phosphate atoms) is slightly wider for Pfu-E10 (13.4 Å) than for RB69 (12.7 Å) but is not as wide as the minor groove in the active site area (15.9 Å) from *Bacillus Stearothermophilus* DNA polymerase. Ternary complexes of A-family DNA polymerases from *B. Stearothermophilus* [28] and T7 phage [29] show the DNA is significantly underwound at the active site, increasing the width of the minor-groove and making it more shallow, possibly allowing more non base-specific contacts in the minor groove to detect Watson-Crick base pair geometry.

### Specificity for Deoxyribonucleotides

DNA polymerases need to distinguish between dNTPs and rNTPs. In the RB69 DNA polymerase the invariant A-motif Tyr 416 provides this selectivity as binding of a rNTP would result in a steric clash between the 2′ hydroxyl and the tyrosine side chain [30]. The RB69 mutant Y416A allows incorporation of rNTP’s. The position of the equivalent ‘steric gate’ tyrosine (residue 410) in the Pfu-E10:DNA complex is consistent with this same mechanism of sugar discrimination. Other RB69 residues involved in RNA discrimination are Asn 564 (Pfu-E10 Asn 492) which co-ordinates the base and sugar of incoming NTP, and the invariant Tyr 567 (Pfu-E10 Tyr 495) that is in van der Waals contact with the steric gate tyrosine. In the Pfu-E10 structure these residues are in similar positions and orientations as in the RB69 polymerase, suggesting they have the same role.

### Structure of the Active Site

The Pfu-E10 structure does not show any density for the catalytically essential divalent metal ions [7] [27], despite the presence of 10 mM Mg^2+^ in the crystallisation medium. In addition, the side chains of the two invariant aspartate residues (Asp 405 and Asp 543) are in the wrong orientation to form a catalytically active conformation. A binary-complex of Phi29 DNA polymerase:DNA [27] also fails to show these catalytic metal ions, and furthermore one of the invariant aspartates is in the wrong orientation for catalysis. It seems probable that the aspartate side chains and metal ions only move into position once the dNTP has bound and the fingers domain has moved to form the dNTP binding pocket.

### Comparison with the Apo Structure

**Figure 7:**
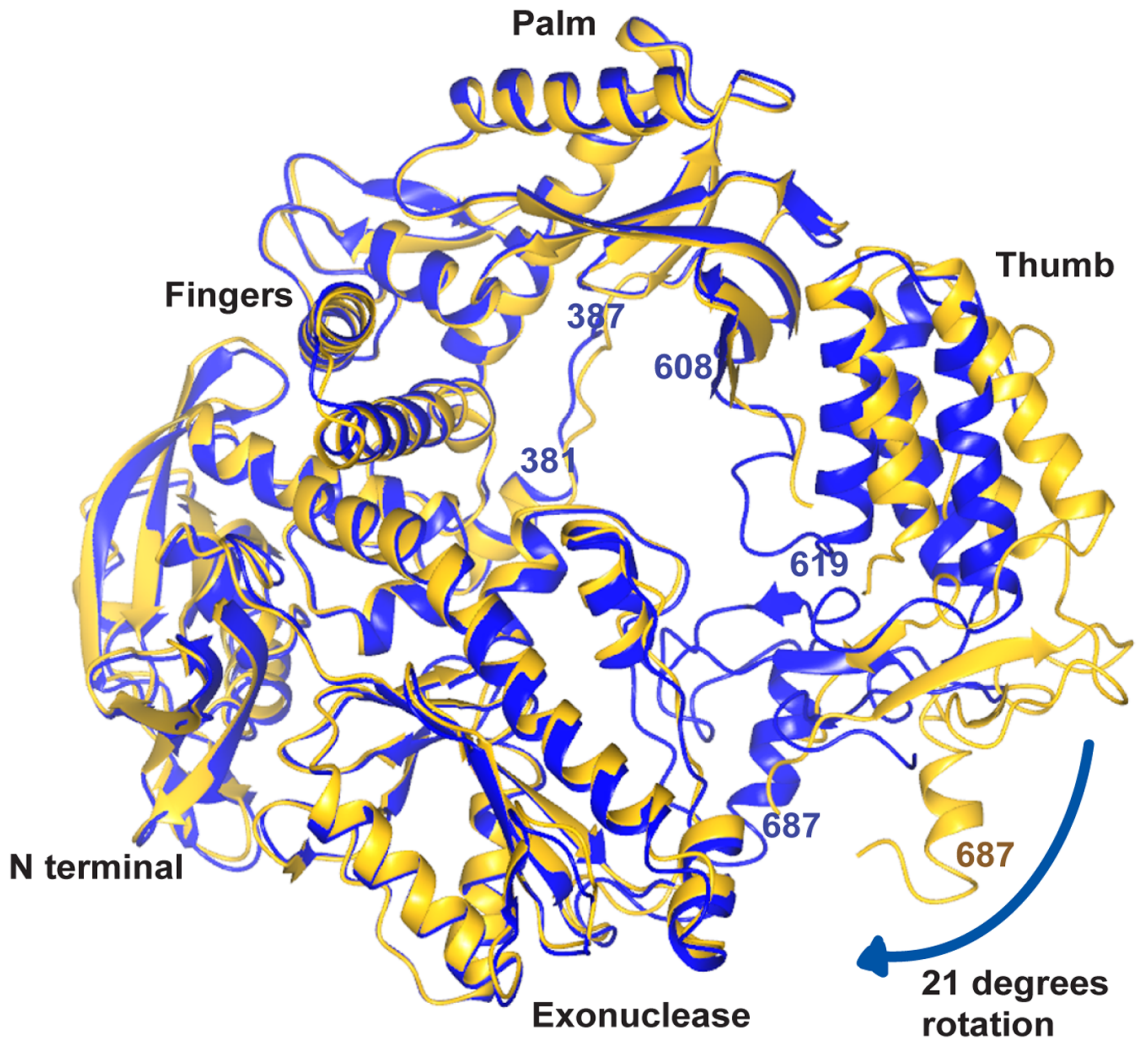
Comparison of the apo and binary structures of the Pfu-E10 polymerase. The apo structure (gold) is shown following superposition of residues in the palm domain on the binary complex (blue). The large movement of the thumb domain is apparent. Residues are labelled in gold for the apo polymerase and in blue for the binary structure.

The N-terminal, exonuclease and linker domains of Pfu-E10 polymerase are largely unchanged upon DNA binding (Figure 7). Within the palm domain, the extended strand containing residues 382–386 lies alongside the DNA template in the binary Pfu-E10:DNA structure. This strand has moved about 2 Å from its position in the apo Pfu-E10 structure to avoid steric clashes with the template. Serine 384 within this strand binds to the template DNA phosphate backbone. The largest difference involves the thumb domain, which has rotated by approximately 21 degrees from the base of the thumb to enclose the DNA and residue Leu 687 in the binary complex has moved 18 Å from its position in the apo form (Figure 7). The loop 608–614 lies against the primer DNA strand, allowing hydrogen bonding between residues in this loop and the DNA. This loop is missing in the native Pfu-pol structure, and has a slightly different conformation in the Pfu-PCNA structure.

### Comparison with the Ternary Complex from RB69

**Figure 8:**
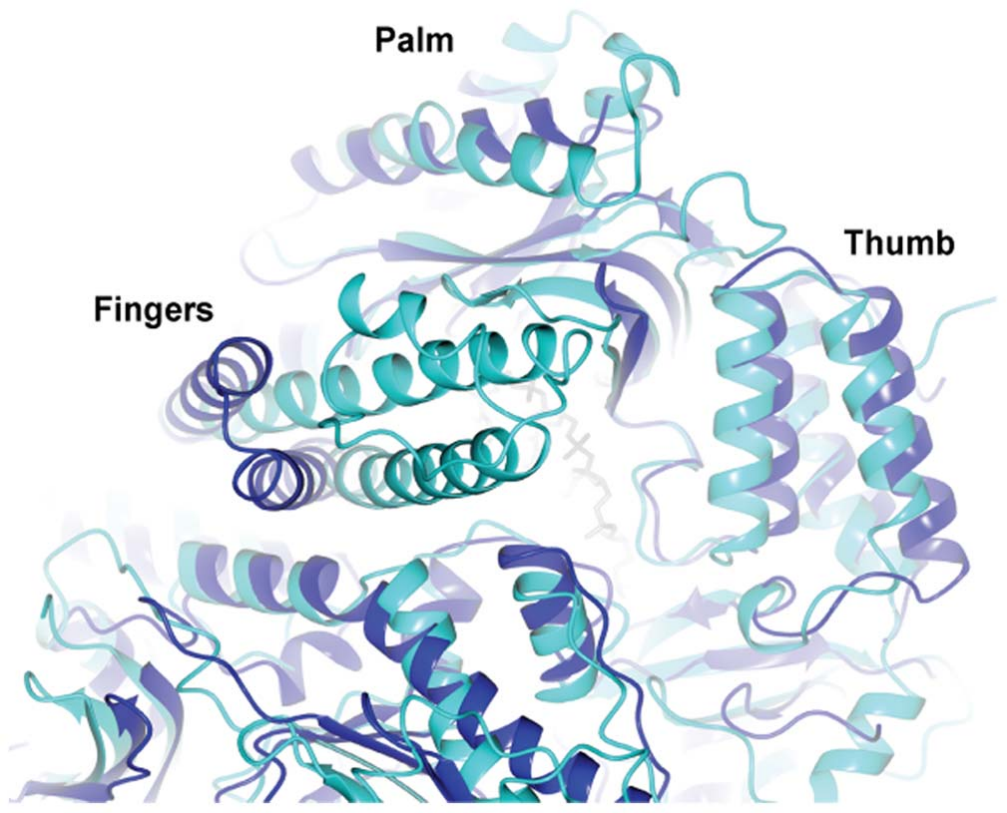
Comparison of the Pfu-E10:DNA binary structure with RB69:DNA:dTTP ternary complex. Superposition of the Pfu-E10:DNA binary structure (blue) onto palm domain residues of the RB69:DNA:dTTP ternary complex (green) (Pfu-E10 residues 384–414, RB69 390–420). DNA from Pfu:E10 complex is shown with grey primer and magenta template. The DNA strands of both structures and many of their domains aligned. The fingers domains show the greatest difference, the RB69 fingers have closed around a dNTP, whereas the Pfu:E10 fingers have not.

Superposition of the Pfu-E10:DNA binary and RB69:DNA:dTTP ternary complex structures [7], based on residues in the palm domains (Pfu-E10 residues 384–414, RB69 390–420) results in an overall rmsd of 1.3 Å (Table 3) and shows that many of the features of these two B-family polymerases are the same (Figure 8), including the position of the DNA duplex. The thumb domains are aligned well, while there are small changes in the orientations of the N-terminal domains (Pfu-E10 residues 1–130, RB69 1–103), the exonuclease domains (Pfu-E10 131–326, RB69 104–340) and the linker regions (Pfu-E10 327–369, RB69 340–380). These differences may be the result of comparing a binary and a ternary complex, or alternatively it is possible that the RB69 polymerase can close more tightly around the DNA than Pfu-E10. The fingers domains however (Pfu-E10 residues 451–500, RB69 471–572) are in quite different orientations. In the RB6 structure the fingers domain closes inwards towards the dNTP, whereas in the Pfu-E10:DNA binary complex the fingers domain remains in the orientation observed in the structure of the apo enzyme, consistent with the absence of a bound dNTP at the active site.

**Table 3:** Structural similarity of Binary E10:DNA complex with RB69 ternary complex.

Comparison with Pfu-E10:DNA complex	Residues aligned^1^	RMSd	% identity
RB69+DNA	31 (Palm region)	1.3	12
RB69+DNA	590 (full Polymerase)	2.8	12

1 Alignment of the Palm region used Pfu-E10 residues 384–414 and RB69 residues 390–420.

### Exonuclease Function

B-family DNA polymerases contain a 3′–5′ exonuclease domain, which removes non-cognate, or non-standard bases. The structure of an editing complex of RB69 polymerase was determined [31] showing that the thumb domain rotates, moving the primer 3′ terminus away from the active site to the exonuclease domain for excision. The DNA remains attached to the thumb tip in the editing complex and RB69 loop residues 784–790 (equivalent to residues 666–675 in Pfu-E10) maintain contacts to the phosphates of the primer strand.

An editing complex of Tgo polymerase was determined with DNA containing uracil bound at the exonuclease site [16]. Compared with the apo Tgo structure, the thumb domain has rotated about a hinge loop region of residues 666–677 that lie alongside the primer. Residues Thr 667, Arg 668, Tyr 673 and Lys 674 of Tgo polymerase interact with the phosphate backbone in this editing complex.

Very recently, structures of editing complexes of another archaeal polymerase B, from *Pyrococcus abyssi*, have been reported [17], with either a deaminated base in the template, or mismatched bases between the primer and template. The binding interactions with the thumb tip include the four interactions seen in the Tgo editing complex, and also DNA phosphate interactions with Ala 675 and His 679.

It was not known if archaeal polymerases would maintain these phosphate contacts during the switch from polymerising to editing mode and back again. However, the equivalent loop in Pfu-E10:DNA binary complex (residues 667–678) lies alongside the primer in the same manner. The structure shows that the Pfu-E10 residues Thr 668, Arg 669, Tyr 674, Lys 675 and Ala 676 interact with the phosphate backbone in the same way as the equivalent Tgo and *P.abyssi* polymerase residues in the editing complexes. It is likely that the entire region maintains the phosphate contacts with the primer strand as it rotates between the binary complex form and editing mode.

### Binding of dCTP and Cy5-dCTP to Pfu-E10:DNA

Extensive attempts were made to produce a ternary complex of Pfu-E10:DNA:dCTP, both by co-crystallisation of the enzyme, primer and template in the presence of 1 mM, 10 mM and 20 mM dCTP, and also by soaking 20 mM dCTP into binary complex crystals. However, the structures derived from diffraction data obtained from these co-crystallised or soaked crystals displayed no evidence for binding of the trinucleotide. In addition, the fingers domain was still in the open conformation observed in the binary complex, rather than displaying the significant conformational change that is normally associated with formation of a ternary complex, when the fingers close towards the nucleotide. This provided further evidence that the nucleotide had not bound. Studying the crystal packing of the binary complex structures revealed that the fingers domain is involved in an extensive lattice contact with the fingers domain of a symmetry related molecule. This may explain why the soaking experiments were not successful as the lattice interactions could block the expected conformational change.

To try to incorporate Cy5 labelled dCTP and create a ternary polymerase:DNA:Cy5-dCTP complex, binary Pfu-E10:DNA crystals were soaked in 10 mM Cy5-dCTP which resulted in a blue colouration. It was not possible to perform co-crystallisation studies with the Cy5-dCTP due to the large cost of this compound, however diffraction data were collected from the Cy5-dCTP soaked crystals. The resulting structures revealed that the fingers domain was still in the open binary conformation and no density was visible either for the large Cy5 dye, nor the dCTP.

**Figure 9:**
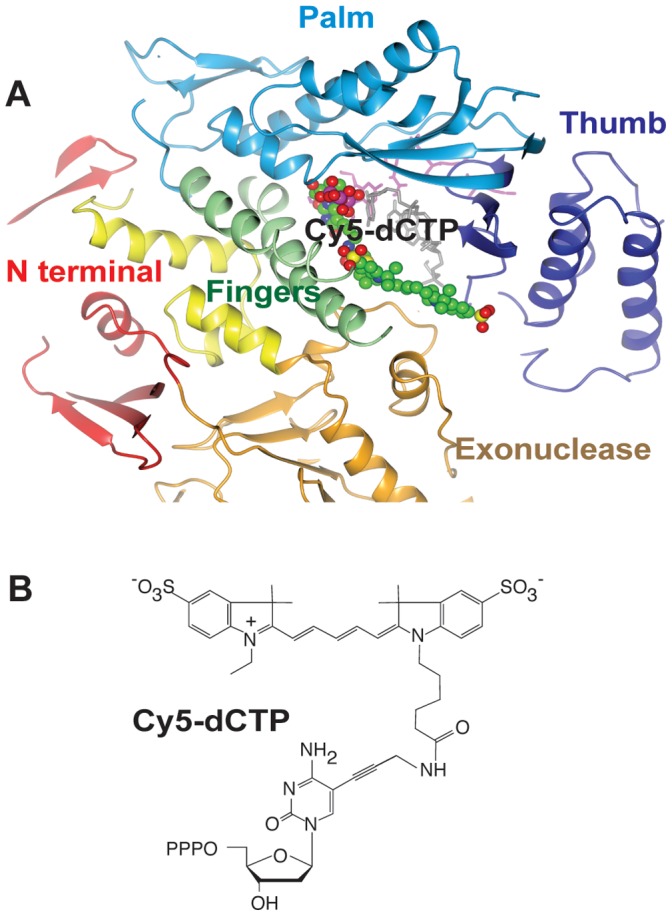
Model of a possible ternary complex of Pfu:E10: DNA:Cy5-dCTP. A). Each domain from the binary PFu:E10:DNA structure was superimposed separately onto the corresponding domain from the RB69:DNA:dTTP ternary complex, and coloured as for Figure 3. A molecule of Cy5-dCTP was then modelled into this with green carbon, red oxygen, blue nitrogen, yellow sulphur and purple phosphorous atoms, to try to understand how such a large molecule could fit into the complex. There appears to be a channel between the Fingers, Thumb and Exonuclease domains into which the large Cy5 molecule could extend. B). Representation of Cy5-dCTP.

In the absence of a ternary complex structure, a putative ternary model for the Pfu-E10:DNA:dCTP complex was created by superimposing each domain from Pfu-E10:DNA binary complex onto the equivalent domain from the RB69:DNA:dTTP ternary complex. The individual domains superimposed more closely than the entire polymerase with rmsd values of 1.3–2.7 Å. A Cy5-dCTP molecule was then modelled into the active site, with the CTP superimposed on the position of the dTTP in the RB69 structure (Figure 9). The modelling suggested that it is possible to accommodate the bulk of the Cy5-dye without any significant impact on the geometry of the active site. While not modelled explicitly, the lack of major groove interactions between the polymerase and the DNA in the Pfu-E10:DNA complex suggests that Cy5-dye modified bases could also be accommodated in the DNA duplex, with the dye molecules located in the major groove of the double stranded DNA.

Ternary structures of KlenTaq (a polA family polymerase) have been solved with modified bases in the active site [32], [33]. In these studies, one of the main residues in the active site that is affected by the incorporation of C5 modified dNTPs is Arg 660 in the B-motif, which is found to be displaced to avoid steric clashes with the bulky C5 substituents. Comparison of the Pfu-E10 binary and the modelled ternary structures with these KlenTaq structures shows that the equivalent Pfu-E10 residue, Lys 485, is already angled away from the DNA, which would allow room for the Cyanine dye modification. Other archael apo and editing polymerase complexes show a variety of positions for this residue.

### The Role of Pfu-E10 Mutations in the Incorporation of Cy5 Modified Nucleotides

In addition to the mutations disabling template uracil binding and exonuclease activity (V93Q, D141A and E143A), Pfu-E10 polymerase has the mutations V337I, E399D, N400D, R407I and Y546H. These additional mutations confer the ability to incorporate cyanine labelled dCTP (Cy3-dCTP or Cy5-dCTP) in place of every dC even in a 70% GC DNA in PCR [19]. The mutations in the A-motif (E399D, N400D and R407I) and the mutation in the C-motif (Y546H) synergistically contribute to this phenotype, with the V337I mutation playing no significant role [19].

Mutations E399D and N400D are located on a beta turn that is remote from the catalytic site (distances of 19.2 Å and 16.8 Å respectively from the alpha carbon of Asp 405) (Figure 3). There is no obvious structural explanation for the effect of these mutations on Cy5-dCTP incorporation, but it is interesting to note that polymerases from the polA family selected for an ability to utilize non-native substrates have mutations at equivalent positions or in the beta strand connecting the beta turn to the active site [18], [34].

Residue 407 is within the active site A-motif (residues 405–412). The alpha carbon is 6.7 Å from that of the invariant Asp 405, and 5.1 Å from the gamma phosphate of a modelled dNTP bound at the active site. The R407I mutation changes a charged arginine to a hydrophobic isoleucine. The smaller side chain of the isoleucine residue might provide greater conformational flexibility helping to accommodate the bulky hydrophobic dye molecule. While the precise role of this residue is currently unclear, it is notable that mutations at Ser 612, the equivalent residue in the active site of polA family polymerases like Taq, enhances incorporation of FITC-12-dATP, another fluorescent dye-labeled nucleotide triphosphate [18]. In FITC-12-dATP the fluorophore is attached to N7 of adenine, and projects into the major groove in a similar way to the cyanine dyes attached to C5 of cytidine in Cy3- and Cy5-dCTP. In a more general way, mutations to the proximal Ile 614 residue in polA polymerases have been found to generally decrease discrimination against non native substrates.

Residue His 546 is on the same beta strand as the C-motif (residues 539–544), which contains the second invariant active site aspartate (Asp 543) (alpha carbon separation of 10.7 Å). 9°N polymerase, which appears to display an increased capacity to incorporate unnatural nucleotide analogues, contains this histidine naturally and is capable of incorporating a limited number of Cy5 dNTP’s. As with the R407I mutation, one could speculate that the smaller histidine side chain may improve accommodation of bulky substrates in the active site.

### Processive Synthesis of Synthetic Genetic Polymers

Previous work [23], [24] has identified a segment of the Tgo DNA polymerase thumb domain (Tgo: E654-T676) as key for the processive synthesis of non-cognate nucleic acid polymers including RNA. The key residue for enabling RNA synthesis in Tgo (Tgo: E664K) is Glu 665 in Pfu-E10. As in Tgo it is located in the thumb domain in proximity to the nascent primer-template duplex without contacting it directly. Other mutations in the same region have been found to be important for the synthesis of synthetic genetic polymers (XNAs) including HNA (1,5 anhydrohexitol nucleic acids), CeNA (cyclohexenyl nucleic acids), LNA (2'-O,4'-C-methylene-β-D-ribonucleic acids; locked nucleic acids), ANA (arabinonucleic acids) and FANA (2'-fluoro-arabinonucleic acid) [35]. These include Glu 610 in Pfu-E10 (Tgo: E609K) which in the Pfu-E10 binary complex structure forms a hydrogen bond with Arg 744 which orientates the guanidine group of Arg 744 to make an interaction with Phosphate10 of the Template strand; Lys 660 (Tgo: K659Q) forms a hydrogen bond with Glu 622 in Pfu-E10; Gln 666 (Tgo: Q665P, H) which binds to Phosphate5 of the primer strand; Arg 669 (Tgo: R668K) which forms a hydrogen bond to Phosphate4 of the primer strand; and Lys 674 (K674R) which bonds to Phosphate3 of the primer strand. Thus several of these residues make direct (or indirect) interactions with the nascent duplex, mainly the primer strand. XNA synthesis is likely to lead to non-cognate helical conformations in the nascent XNA-DNA duplex requiring repositioning and modification of such interactions to enable processive synthesis. However, a more complete understanding of the function of these different residues in the thumb domain and their contribution to DNA (and XNA) synthesis will require a dissection of their individual contributions and a fuller analysis of the effects as performed for Glu 664 in the case of RNA synthesis [24].

### Conclusions

This is the first reported structure of a binary complex of an archael B family DNA polymerase with duplex DNA bound in synthesis mode. This structure has added to the understanding of thermophilic polymerases and allowed comparison with other archael and mesophilic polymerases (*Tgo*, *P.abyssi*, Phi29 and RB69).

Although the mechanistic basis of enhanced Cy5-dCTP incorporation by Pfu-E10 is still unclear, the structures described herein have already provided extremely useful insights in understanding the structural context of mutations in the thumb subdomain, their interactions with the nascent strand and their contribution to the ability of polB family polymerases to synthesize synthetic genetic polymers (XNAs) [35] or long RNAs. We hope that the structures reported here will prove useful to others who are trying to design biotechnologically important polymerases.

## Materials and Methods

### Cloning and Purification

Pfu-E10 Polymerase was cloned into pT7-SC plasmid (United States Biochemicals) and over-expressed with BL21-codon+(DE3)-RIL (Stratagene) *Escherichia coli* cells, induced mid log phase with isopropyl β-D-1-thiogalactopyranoside (IPTG). RIL cells contain tRNAs for rare *E. coli* codons of arginine, isoleucine and leucine and can therefore increase expression of non-*E. coli* proteins. The Pfu-E10 polymerase gene contains 86 of these rare codons (11.1% of the gene) and using this expression system allowed purification of milligrams of protein per litre of cells.

The cells were centrifuged two hours after induction and re-suspended in 50 mM Tris pH 7.4, 1% glucose. This was mixed with 1.25 volumes of 10 mM Tris pH 7.4, 50 mM potassium chloride, 0.5% nonyl phenoxypolyethoxylethanol (NP-40) and 0.1% Triton, and incubated at 70° C for 30 minutes to lyse the cells. The lysate was centrifuged and the supernatant passed through 50 ml DE52 ion exchange media (pre-equilibrated in 20 mM Tris pH 7.4, 50 mM sodium chloride, 10% glycerol), and collected. The flow-though was loaded onto a 50 ml column of heparin-sepharose (pre-equilibrated with the same buffer) and washed to remove any remaining contaminants. The purified Pfu-E10 was eluted with 600 mM sodium chloride, 20 mM Tris, 10% glycerol.

### Protein Concentration Normalization

We have previously demonstrated that E10, when normalised by dNTP incorporation activity, outperforms Pfu(exo-) in the incorporation of dCTP-Cy5 [19] Here we took an alternative approach, normalising protein concentration to demonstrate that the E10 polymerase described outperforms the wild-type from which it was derived (Pfu (exo-)); we worked under the assumption that the active polymerase fraction in the commercial Pfu(exo-) (Agilent, USA) was comparable to the E10 polymerase we isolated as described above.

Briefly, a serial dilution of bovine serum albumine (BSA; NEB, USA) was used to create a standard curve against which the polymerase concentrations could be estimated and used to normalise the samples (Figure S2A). Protein samples were adjusted to 5 µl with water, and 2 µl of 4× NuPage® LDS Sample Buffer (Invitrogen, UK) and 1 µl of 100 mM DTT (Sigma-Aldrich, UK) were added to each sample. 10 µl of the Color Plus prestained protein marker (NEB, USA) were used as a molecular weight marker. Samples were heated to 85°C for 5 minutes prior to being separated by electrophoretic mobility in a NuPAGE 4–12% Bis-Tris polyacrylamide gel (Invitrogen, UK) running 1× NuPAGE MOPS SDS Running buffer (Invitrogen, UK).

Polyacrylamide gel was washed in deionised water (ddH_2_O) and incubated in 7.5% acetic acid (Sigma-Aldrich, UK) with 1× SYPRO Orange (Invitrogen, UK) for approximately 10 minutes at room temperature. The stained gel was washed briefly in clean 7.5% acetic acid prior to visualization by fluorescence using a Typhoon TRIO variable mode imager (GE Healthcare UK Ltd., UK) –580 nm emission filter, 400 PMT and 488 nm excitation.

Samples were analysed using ImageJ software, to determine their signal strength and allow protein concentrations to be estimated. Based on the standard curve obtained with BSA, polymerase concentrations were estimated and used in their normalization. Pfu(exo-) concentration was estimated to be around 0.2 mg ml^−1^ and E10 was diluted 4.5-fold in storage buffer (10 mM Tris-HCl pH 7.4, 100 mM KCl, 1 mM DTT, 0.1 mM EDTA). Protein concentration normalisation was confirmed by PAGE (Figure S2B).

### Polymerase Extension Assays

A challenging template requiring seven sequential incorporations was selected to maximise discrimination between wild-type and engineered polymerases (tempG: 5′-TTCGGTAATCGATCGGGGGGGATCTGGCAAACGCTAATAAGG).

Primer extension reactions were carried out in 10 µl buffer containing 100 nM primer (fd: 5′-FAM-CCCCTTATTAGCGTTTGCCA), 300 nM template (tempG), 10 µM of dATP, dGTP and dTTP (GE Healthcare UK Ltd., UK), 10 µM of Cy5-dCTP (GE Healthcare UK Ltd., UK) and 15 ng of polymerase (or 0.06U of Pfu(exo-)). Cy5-labelled dCTP was replaced with ddH_2_O (negative control) or unlabelled dCTP (positive control) as required. The primer was labelled with FAM (fluorescein) so that it could be visualised by the Typhoon scanner regardless of any cyanine dye incorporation.

Reactions were annealed (2 minutes at 95°C and cooled at 0.1°C s^−1^ to 4°C) and polymerase added while reactions were kept on ice. Reactions were transferred to a pre-heated PCR block and incubated at 50°C or 60°C for varying periods of time. Reactions were quenched by adding 30 µl of quenching buffer (98% formamide, 10 mM EDTA). Controls were carried out using the longest time points investigated (15 minutes for reactions carried out at 60°C and 1 hour for reactions carried out at 50°C).

Extension products were separated by denaturing PAGE (8 M Urea, 1× TBE, 20% acrylamide gels) and visualised with the Typhoon TRIO variable mode imager (GE Healthcare UK Ltd., UK) using standard FAM or Cy5 settings, to visualise either the fluorescein or the cyanine dyes.

### Quantifying Polymerase Extension

As the template used in the polymerase extension assays was designed to provide a significant challenge for dCTP-Cy5 incorporation, polymerase activity was quantified by the ability of the polymerase to incorporate seven consecutive Cy5-dCTP in a template-dependent manner.

Extension products separated by PAGE were analysed using ImageJ software, to determine the signal strength of the fragments extended beyond the seven incorporations as a fraction of the total signal detected per reaction. Quantification was carried out on two independent experiments.

### Crystallisation

Pfu-E10 protein was exchanged into 20 mM Tris pH 7.4, 50 mM sodium chloride, 10% glycerol buffer and concentrated to 20 mg/ml in a Sartorius Vivaspin unit. Crystallisation trials were set up with an Innovodyne Screenmaker 96+8 crystal robot, with 10–15 mg/ml Pfu-E10 protein in 96-well plates. The conditions were optimised in 24-well, hanging-drop plates with 1 µl protein mixed with 1 µl well buffer. The best crystals were grown using well conditions of 0.08 M sodium cacodylate pH 6.5, 0.14 M magnesium acetate, 12% PEG 8000, 20% glycerol, and were flash frozen in liquid nitrogen. Complexes of Pfu-E10 with a number of different DNA constructs were prepared in dilute solution (20 mg protein, 100 mg DNA in 3 ml buffer), in 20 mM Tris, 50 mM sodium chloride, 5 mM magnesium chloride, 5% glycerol and 1 mM dCTP, with a molar ratio of 1∶5 protein to DNA. The complexes were incubated overnight at 4°C and concentrated to approximately 12.5 mg/ml protein with Vivaspin concentrator units. The complexes were screened for crystallisation with the Innovodyne robot. 100 nl of protein:DNA complex (at 10 mg/ml protein) were mixed with 100 nl well buffer in 96-well plates. Conditions were optimised by hand in 24 well plates, using 2 ml drops.

The best crystals were obtained using the template 5′ACGGGTAAGCA3′, and primer 5′TGCTTAC(DOC) 3′ oligonucleotides, where di-deoxy cytidine (DOC) was used as the last primer base to stall the enzyme and prevent further polymerisation. The crystals grew in 0.1 M sodium cacodylate pH 6.5, 0.1 M sodium citrate, 30% isopropanol and 10 mM dCTP. Cryoprotection was achieved by quickly sweeping the crystals through 10% isopropanol, 10% ethylene glycol, 25% glycerol, before flash-freezing in liquid nitrogen.

### Structure Determination and Refinement

Diffraction data for the apo enzyme were collected at beamline ID 14-2 at the European Synchrotron Radiation Facility (ESRF), Grenoble from cryo-cooled crystals (100 K) using an ADSC Q210 CCD detector (λ = 0.9334 Å). Diffraction data from cryo-cooled crystals (100 K) of the binary complex were measured at beamline IO3 at the Diamond Light Source (DLS), Harwell, using an ADSC Q315 CCD detector (λ = 0.9763 Å). Diffraction images were processed using iMosflm [36] and SCALA [37] . The apo pfu-E10 structure was solved by molecular replacement with AMORE [38] using Tgo DNA polymerase [12] (PDB code 1TGO) as the molecular replacement model. Phenix.Xtriage [39] indicated that the crystal was highly twinned (twinning fraction 0.46), with a twin law of *l* –*k h*. The model was refined against data to 2.4 Å resolution initially using Phenix.refine [39] and then with Refmac [40] using twin refinement and applying non crystallographic symmetry restraints between the two copies in the crystallographic asymmetric unit. Manual rebuilding was carried out using COOT [41].

The Pfu-E10:DNA binary complex structure was solved by molecular replacement with the program AMORE, using the apo Pfu-E10 structure as the starting model. Refinement against data to 2.9 Å resolution was carried out initially with Phenix.refine [39] and then with Refmac [40]. Manual building was performed using COOT [41]. Both structures were validated using the program Molprobity [42]. Superposition analysis of structures was undertaken with the CCP4 program SUPERPOSE using the entire polypeptide chain unless otherwise stated, and sequence alignments were carried out using Clustal W.

Coordinates and structures factors have been deposited with the Protein Data Bank, with PDB codes 4AHC (apo enzyme) and 4AIL (binary complex).

### Note Added in Proof

While this manuscript was under review, Bergen *et al* [43] reported structural analyses of wild-type KOD and 9°N polymerases in the binary form with DNA in the active site. Both KOD and 9°N are closely related to Pfu (80% and 81% sequence identity respectively) but do not display the fluorescent dye incorporation phenotype of the mutant Pfu polymerase E10 described in this paper. Nevertheless, their structural analyses confirm our findings on the nature of the interactions between the polymerase and the primer and template strands of the duplex.

## Supporting Information

Figure S1 **Polymerase activity at 50°C.** A) Primer extension time course comparing wild-type Pfu(exo-) and engineered Pfu-E10 polymerases at 50°C. Extension times are shown in minutes. Extension products used to quantify extension beyond the seven consecutive dCTP-Cy5 incorporations (C_7_ challenge) are highlighted in red – see Materials and Methods for details. B) Fraction of the primers extended beyond the C_7_ challenge for both tested polymerases – results are shown for two independent experiments.
(TIF)

Figure S2 **Protein concentration normalization.** A) PAGE was used to estimate polymerase concentration against a standard curve generated from bovine serum albumin (BSA). Based on the estimates obtained, protein concentration was normalised and subsequently checked by PAGE (B).
(TIF)
