# Supplementary figures and images for: Structures of an Apo and a Binary Complex of an Evolved Archeal B Family DNA Polymerase Capable of Synthesising Highly Cy-Dye Labelled DNA
Source: PLoS One. 2013 Aug 5;8(8):e70892. doi: 10.1371/journal.pone.0070892 (PMC3733885; doi:10.1371/journal.pone.0070892)

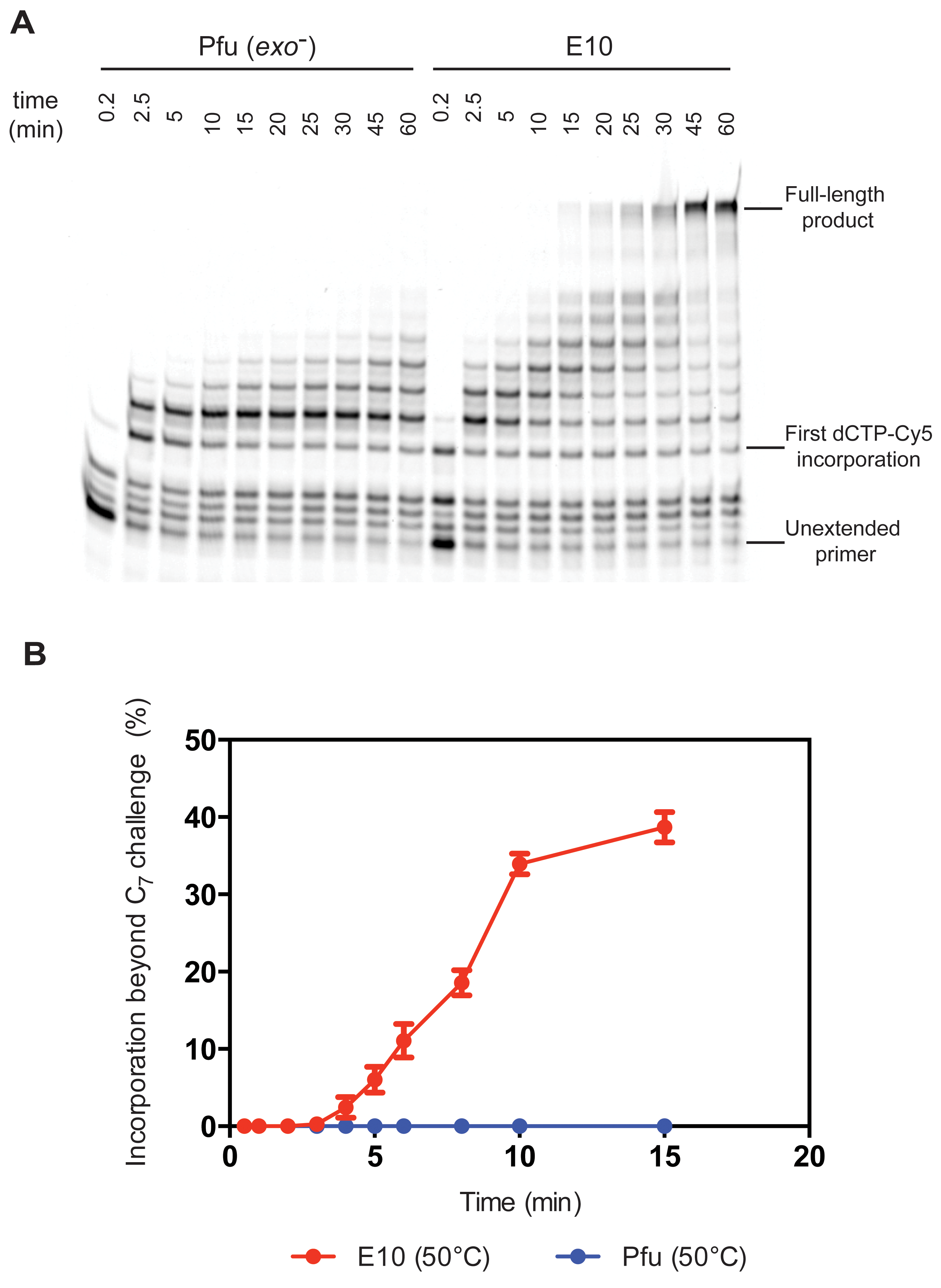

Supplement: Figure S1 — Polymerase activity at 50°C. A) Primer extension time course comparing wild-type Pfu(exo-) and engineered Pfu-E10 polymerases at 50°C. Extension times are shown in minutes. Extension products used to quantify extension beyond the seven consecutive dCTP-Cy5 incorporations (C7 challenge) are highlighted in red – see Materials and Methods for details. B) Fraction of the primers extended beyond the C7 challenge for both tested polymerases – results are shown for two independent experiments. (TIF) [file pone.0070892.s001.tif]

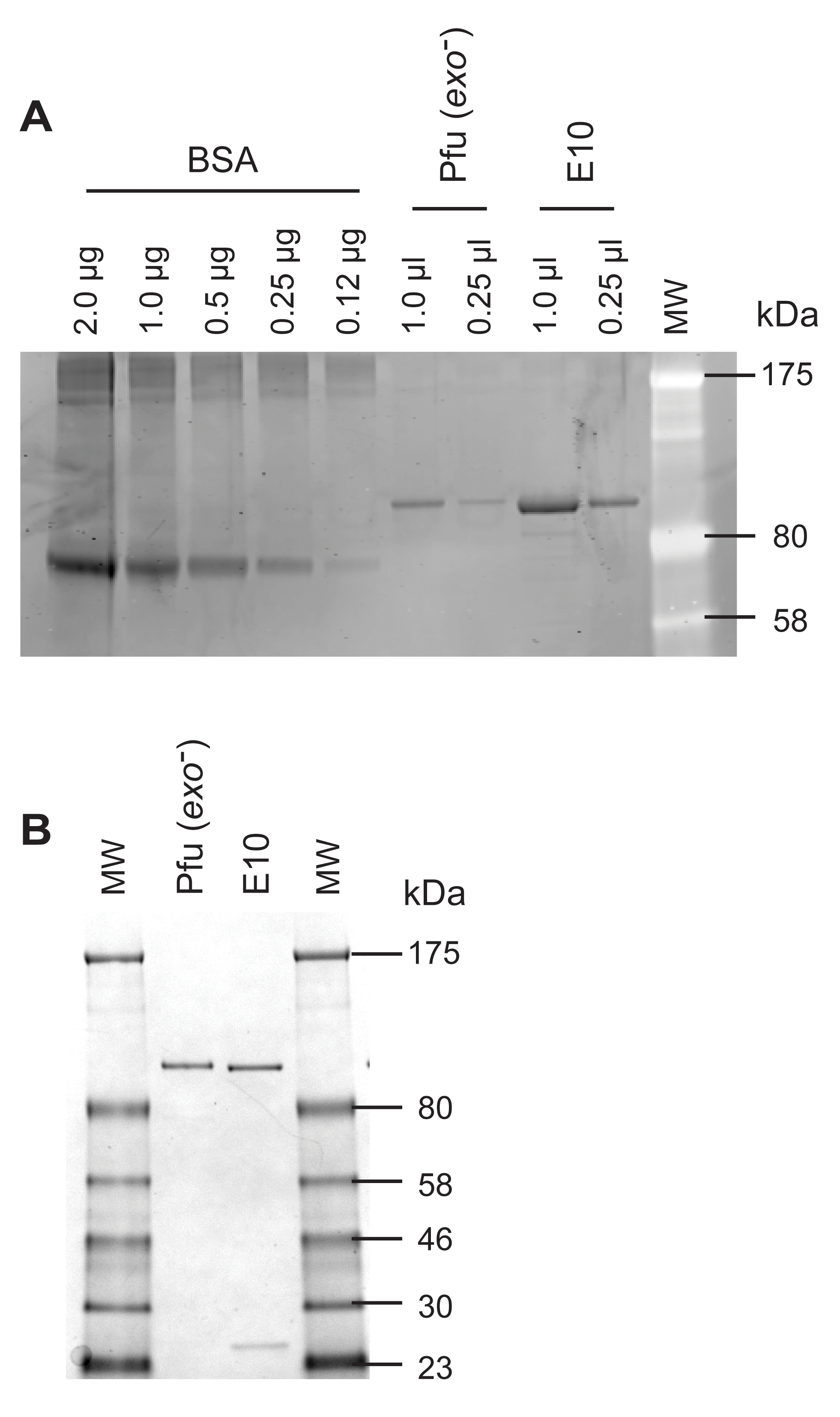

Supplement: Figure S2 — Protein concentration normalization. A) PAGE was used to estimate polymerase concentration against a standard curve generated from bovine serum albumin (BSA). Based on the estimates obtained, protein concentration was normalised and subsequently checked by PAGE (B). (TIF) [file pone.0070892.s002.tif]
